# Normative weight-adjusted models for the median levels of first trimester serum biomarkers for trisomy 21 screening in a specific ethnicity

**DOI:** 10.1371/journal.pone.0182538

**Published:** 2017-08-03

**Authors:** Ounjai Kor-anantakul, Thitima Suntharasaj, Chitkasaem Suwanrath, Tharangrut Hanprasertpong, Savitree Pranpanus, Ninlapa Pruksanusak, Suthiraporn Janwadee, Alan Geater

**Affiliations:** 1 Department of Obstetrics and Gynaecology, Faculty of Medicine, Prince of Songkla University, Hat Yai, Songkhla, Thailand; 2 Epidemiology Unit^2^, Faculty of Medicine, Prince of Songkla University, Hat Yai, Songkhla, Thailand; Clinical Institute of Medical Genetics, SLOVENIA

## Abstract

**Objective:**

To establish normative weight-adjusted models for the median levels of first trimester serum biomarkers for trisomy 21 screening in southern Thai women, and to compare these reference levels with Caucasian-specific and northern Thai models.

**Methods:**

A cross-sectional study was conducted in 1,150 normal singleton pregnancy women to determine serum pregnancy-associated plasma protein-A (PAPP-A) and free β-human chorionic gonadotropin (β-hCG) concentrations in women from southern Thailand. The predicted median values were compared with published equations for Caucasians and northern Thai women.

**Results:**

The best-fitting regression equations for the expected median serum levels of PAPP-A (mIU/L) and free β- hCG (ng/mL) according to maternal weight (Wt in kg) and gestational age (GA in days) were: $\text{median}\;\text{PAPP}-\text{A}={\text{e}}^{\left[8.4454\;-\;0.01950\;\left(\text{Wt}-55\right)+\;0.05747\;\left(\text{GA}-87\right)\right]},$ and $\text{median}\;\text{free}\;\beta -\text{hCG}={\text{e}}^{\left[3.6409\;-\;0.01703\;\left(\text{Wt}-55\right)–\;0.03345\;\left(\text{GA}-87\right)\right]}.$ Both equations were selected with a statistically significant contribution (p< 0.05). Compared with the Caucasian model, the median values of PAPP-A were higher and the median values of free β-hCG were lower in the southern Thai women. And compared with the northern Thai models, the median values of both biomarkers were lower in southern Thai women.

**Conclusion:**

The study has successfully developed maternal-weight- and gestational-age-adjusted median normative models to convert the PAPP-A and free β-hCG levels into their Multiple of Median equivalents in southern Thai women. These models confirmed ethnic differences.

## Introduction

The first trimester combined screening test has been proven to be an effective prenatal screening method for trisomy 21, with a high detection rate of 85–95% and a false positive rate of only 5% [1–3]. The method combines maternal age, measurement of fetal nuchal translucency thickness (NT) and serum biochemical markers, which include pregnancy-associated plasma protein-A (PAPP-A) and free β-human chorionic gonadotropin (β-hCG). The interpretation of these biochemical markers is routinely based on the Caucasian-specific model; however, several studies observed that the median values of these markers are higher among Asian populations relative to other ethnicities [4, 5]. For example, a comparable study by Leung et al [6] found that Chinese women have a higher level of both serum markers in the maternal circulation than Caucasian women. To ensure that screening is effective, population-specific models for median levels of PAPP-A, and free β-hCG should be developed to provide more accurate risk calculations [6, 7].

This study aimed to establish reference ranges for median levels of first trimester serum PAPP-A and free β-hCG in southern Thai women adjusted by maternal weight, and to compare these reference levels with Caucasian- specific and northern Thai models.

## Materials and methods

This was a cross sectional study conducted at Songklanagarind Hospital, a university hospital in southern Thailand, from 2012 to 2014. Serum PAPP-A and free β-hCG concentrations were determined for 1,359 normal singleton pregnant women between 77 and 93 days’ gestation. The inclusion criteria comprised southern Thai women, normal uncomplicated pregnancy, reliable gestational age from the last menstrual period and real time ultrasonography examination of crown-rump length using GE Voluson 730 Expert or E8 (GE Health Care, Waukesha, WI, USA). Demographic characteristics were recorded at the time of blood sampling, including maternal age, parity and gravidity, maternal body weight, and ethnic origin. The exclusion criteria were assisted reproduction conception, known medical maternal complication, smoking, multifetal pregnancy, gestational diabetes, pregnancy-induced hypertension, and/or confirmed chromosome abnormalities. The study was approved by the Ethics Committee, Faculty of Medicine, Prince of Songkla University, Thailand. Written informed consent was obtained from all individual participants included in the study.

The blood samples were immediately transported to the laboratory and the serum was separated by centrifugation. The serum markers were determined using electro-chemo-luminescence immunoassay on an MODULAR ANALYTICS E170 or cobas e 411 analyzer from Roche Diagnostics, Germany. Data on pregnancy outcome were obtained from either our maternity data base for those who delivered in our hospital or via telephone calls to the mothers themselves.

### Statistical analysis

The overall distributions of serum PAPP-A and free β-hCG with respect to gestational age were examined by plotting the reported levels of each marker against gestational age. Logarithmic transformation of each measure produced a more symmetrical distribution and was used in subsequent analyses. The predicted median values of serum PAPP-A and free β-hCG according to gestational age were then estimated using quantile regression and compared with median values for each gestational day from the raw data by plotting both against gestational age.

To take account of the effect of maternal weight, further quantile regression models were constructed in which both gestational age and maternal weight (both centralized by subtracting the respective mean value of the dataset) were included as predictor variables. Deviations from the log-linear relationship were explored by the addition to the model of quadratic terms for each predictor. The best fitting model for each of PAPP-A and free β-hCG was selected based on each predictor term making a statistically significant contribution (p<0.05) to the fit of the model. Surface plots depicting the predicted median levels of serum PAPP-A and free β-hCG against gestational age and maternal weight were constructed and compared with similar surfaces based on published data for Caucasian [8] and northern Thai populations [9]. Multiple of the median (MoM) values for serum PAPP-A and free β-hCG in our subjects were then calculated based on our predicted median values for southern Thai women using the appropriate gestational age and maternal weight, and then compared with the MoM values that would have been obtained using the corresponding predicted median values for Caucasian and northern Thai women.

## Results

**Fig 1:**
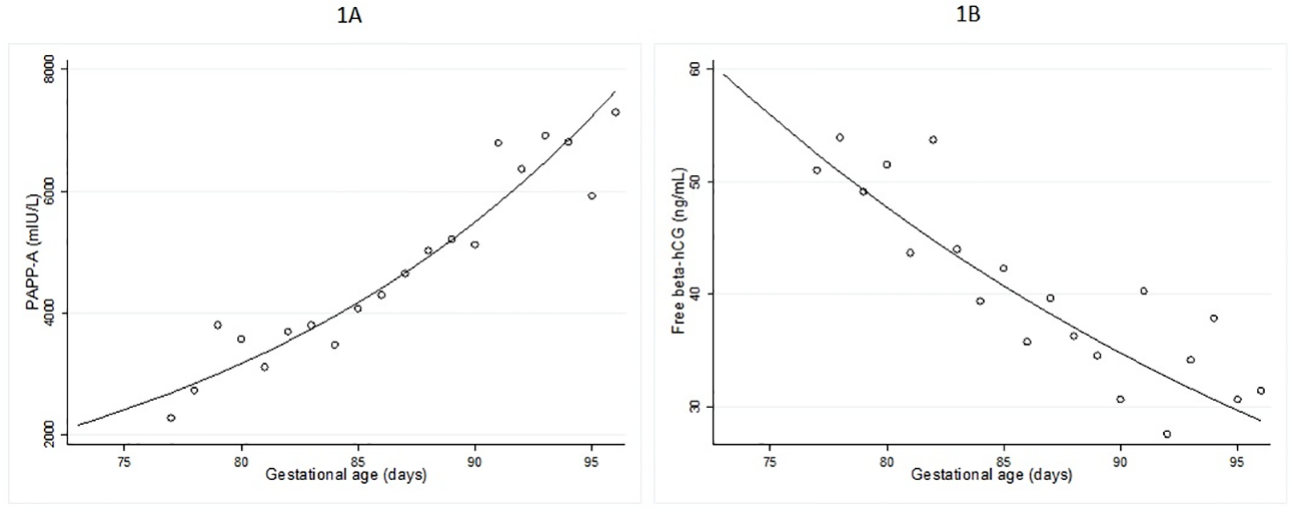
Median values of PAPP-A (mIU/L) [A] and free β -hCG (ng/mL) [B] according to gestational age (days). The circles represent the daily median value of the raw data. In each graph, the line is the predicted median from quantile regression against gestational age. The regression line is not adjusted for maternal weight.

During the study period, 1,359 women were enrolled in the screening program but 209 pregnancies were not included in this analysis due to maternal complication (e.g. preeclampsia, preterm labor, gestational diabetes mellitus, and other medical complications) or neonatal complication (e.g. needing to be supported in the neonatal intensive care unit, proven congenital anomaly), leaving a total of 1,150 pregnancies (85%) with normal fetal outcome as documented by the neonatologists to be included in the analysis. Four women had a trisomy 21 fetus detected by karyotyping. The median maternal age at the time of screening was 31 years (interquartile range 15–44 years); 16% of the women were 35 years of age or older. All cases were urban residents in southern Thailand. The characteristics of the study population are summarized in Table 1. Crude median values of serum PAPP-A and free β-hCG of women with normal fetuses for each gestational day, together with the predicted medians ignoring maternal weight are shown in Fig 1A and 1B. It can be seen that PAPP-A increases and free β-hCG decreases with increasing gestational age throughout the period under study.

**Table 1:** Demographic characteristics of the study population (1,150 women).

Parameter	Median (range) or n (%)
1. Maternal age at time of blood sampling, years	31 (15–44)
2. Maternal age ≥ 35 years at delivery	186 (16)
3. Maternal weight at time of blood sampling, kg	55 (37–101)
4. Parity	
• 0	653
• ≥ 1	497
5. Gestational age at screening, days	87 (73–96)
• 70–76	2 (0.2)
• 77–83	213 (18.5)
• 84–90	713 (62.0)
• 91–97	222 (19.3)
6. Crown-rump length, mm	60.5 (35.6–77)
7. Gestational age at delivery, weeks	38 (32–42)
8. Birth weight, g	3,184 (2,048–4,870)

The inclusion of quadratic terms in the quantile regression models including both gestational age and maternal weight as predictors did not improve the fit either for PAPP-A or for free β-hCG, and these terms were therefore omitted. Thus, the logarithmically transformed median PAPP-A increased and median free β-hCG decreased linearly with increasing gestational age and both decreased linearly with increasing maternal weight.

The best-fitting regression equations for the expected median serum levels of PAPP-A (mIU/L) and free β-hCG (ng/mL) according to maternal weight (Wt in kg) and gestational age (GA in days) in our study population of normal southern Thai pregnant women were:

$$\text{Median}\;\text{PAPP}-\text{A}={\text{e}}^{\left[8.4454\;-\;0.01950\;\left(\text{Wt}-55\right)+\;0.05747\;\left(\text{GA}-87\right)\right]}$$

$$\text{Median}\;\text{free}\;\beta -\text{hCG}={\text{e}}^{\left[3.6409\;-\;0.01703\;\left(\text{Wt}-55\right)–\;0.03345\;\left(\text{GA}-87\right)\right]}.$$

**Fig 2:**
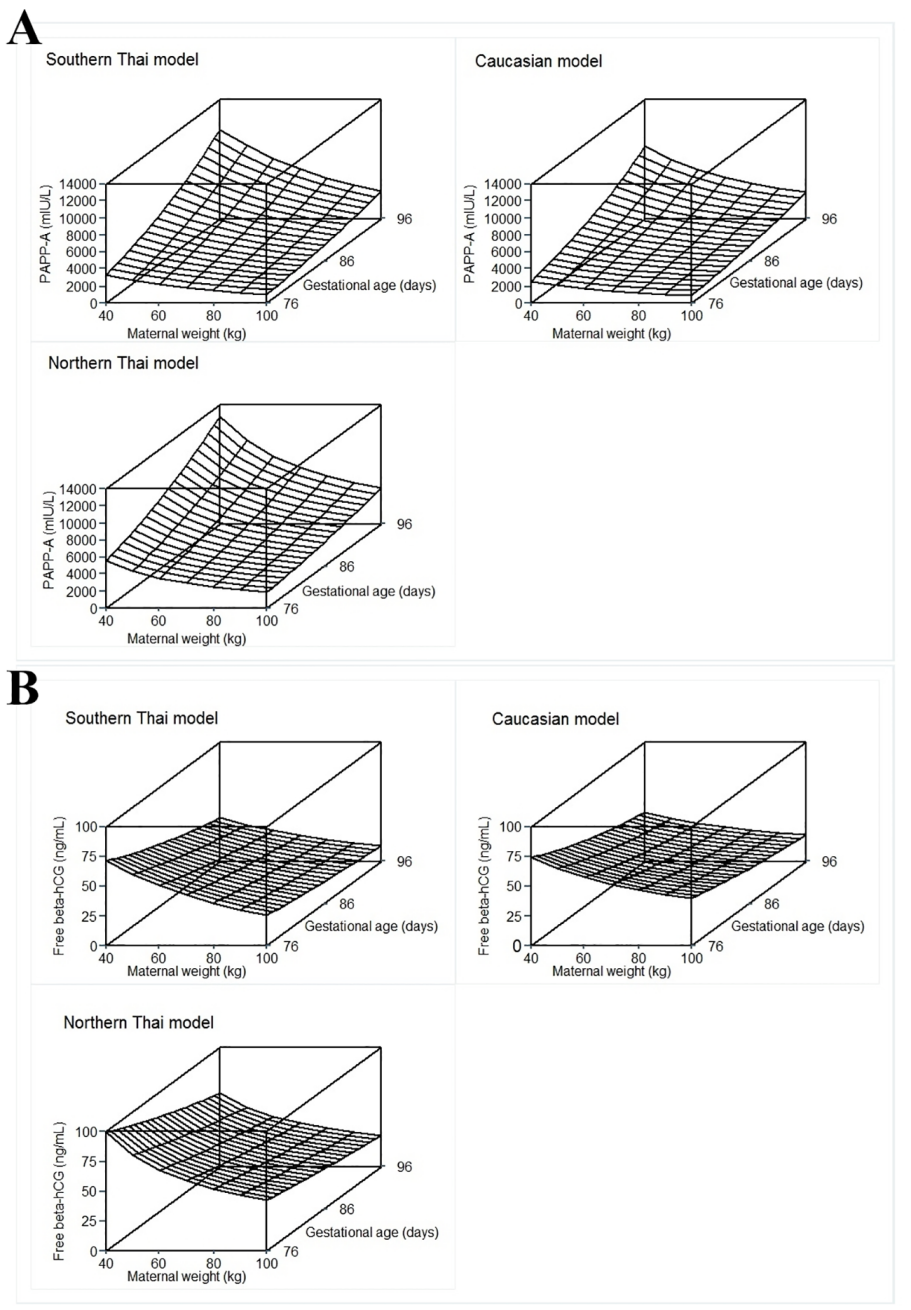
Surface of predicted median PAPP-A level (mIU/L) [A] and predicted median free β -hCG (ng/mL) [B] obtained from quantile regression models against gestational (days) and maternal weight (kg). In each figure, the left upper side is the result of the quantile regression using the southern Thai data, the right upper side is based on the published equation for median value for a Caucasian population [8], and the left lower side is based on the published equation for a northern Thai population [9].

The relationships of predicted median serum PAPP-A and free β-hCG with gestational age and maternal weight for the respective models for the three populations are shown by the surface plots in Fig 2A and 2B. As can be seen, the median values of PAPP-A are higher in the southern Thai population than in the Caucasian population especially at older gestational ages and lower maternal weights. Moreover, these median values are lower than from the northern Thai population. Furthermore, the median values of free β-hCG are lower in the southern Thai population than in the Caucasian and northern Thai populations at all gestational ages but especially at higher maternal weights within the ranges considered here.

**Fig 3:**
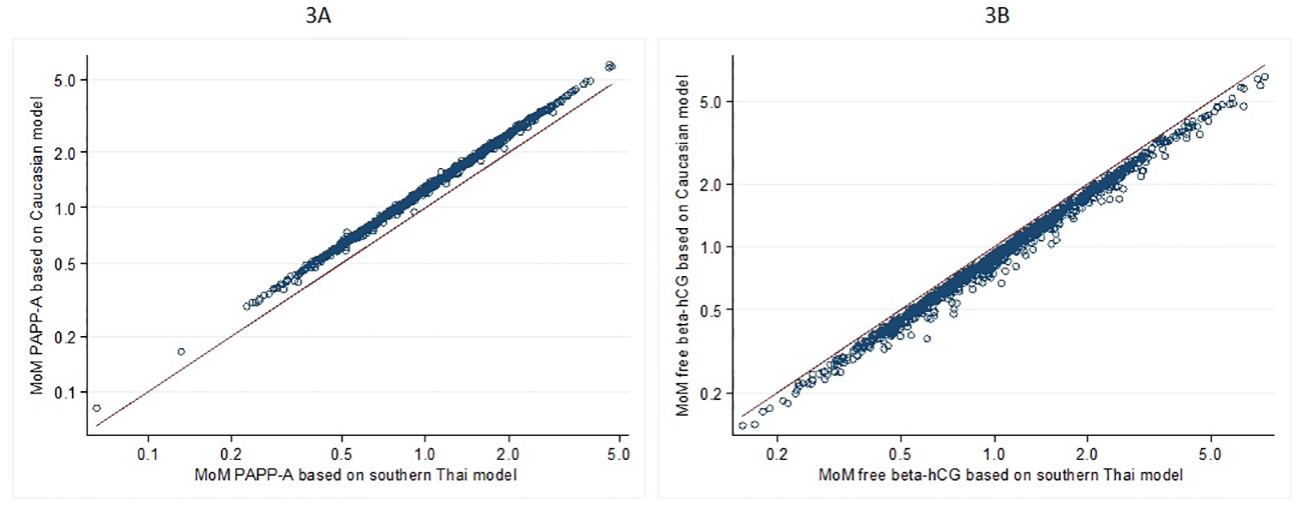
The relationship between the values of MoM for PAPP-A [A] and free β-hCG [B] using the southern Thai median equation from this study on the X-axis, and the Caucasian equation [8] on the Y-axis (circles). The line represents the line of equity, i.e., marking the same values on both X and Y-axes.

**Fig 4:**
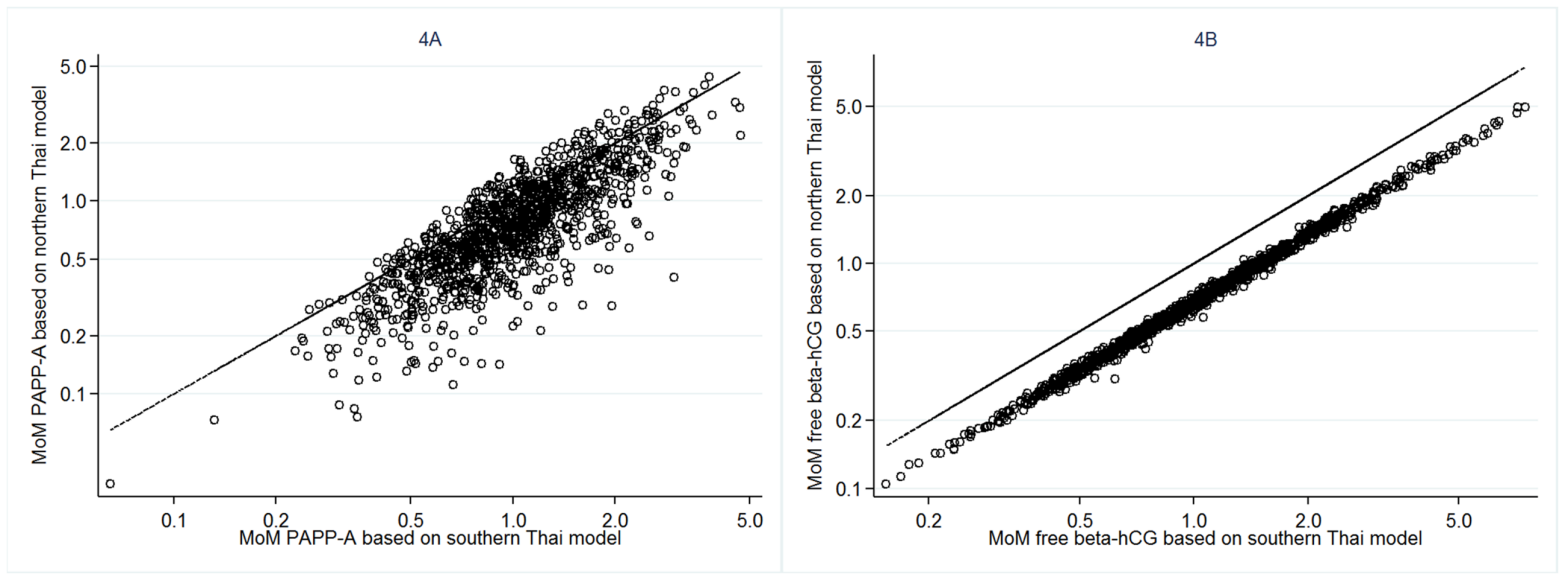
The relationship between the values of MoM for PAPP-A [A] and free β-hCG [B] using the southern Thai median equation from this study on the X-axis, and the northern Thai equation [9] on the Y-axis (circles). The line represents the line of equity, i.e., marking the same values on both X and Y-axes.

The relationships between the MoM values of serum PAPP-A and free β-hCG for our southern Thai women predicted medians and the MoM values that would be obtained using the Caucasian predicted medians are shown in Fig 3A and 3B. Estimations using the Caucasian model would result in higher values for MoM of serum PAPP-A and lower values for MoM of serum free β-hCG compared with this study model. Similary, Fig 4A and 4B show the relationship between the MoM values of serum PAPP-A and free beta hCG based on the southern Thai model and the northern Thai model. Estimations using the northern Thai model would result in lower values for both serum biomarkers.

The four women with fetal trisomy 21 presenting during the same period of data collection had the weight-corrected values of MoM PAPP-A and MoM free β -hCG by our model compared with the Caucasian model as shown in Table 2.

**Table 2:** MoM PAPP-A and MoM free β -hCG by our model compared with the Caucasian model in the four women with fetal trisomy 21.

no	MoM PAPP-A		MoM free β -hCG	
	southern Thai	Caucasian	southern Thai	Caucasian
1	0.199	0.249	0.904	0.756
2	0.582	0.745	1.941	1.780
3	0.448	0.572	2.670	2.462
4	0.659	0.801	3.012	2.366

## Discussion

This study provides maternal-weight- and gestational-age-adjusted median normative models for use in first-trimester screening programs for southern Thai women. In this population, applying the Caucasian model, the MoM of PAPP-A would be overestimated whereas the MoM of free β-hCG would be underestimated, applying the northern Thai model, MoM of both markers would be underestimated. In both cases, using either model could lead to an inaccurate estimation of the risk of Down syndrome.

Usually the concentration of serum PAPP-A increases, and free β-hCG decreases, with advancing gestational age, and both decrease with increasing maternal weight [10]. Our study found the same tendencies. Log-linear regression and exponential equation models were used in this study as they showed the best fit for both gestational days and maternal weight. Similar to a previous study that reported maternal weight correction in the first trimester, screening can be best achieved using the log-linear procedure [11]. Although including a weight correction parameter seems to have an almost negligible impact on true positive rates and false positive rates, for an individual woman, weight correction can have a non-negligible impact on calculating specific risk [12]. Thus, to improve the accuracy of screening tests for individual specific risk, the MoM values of serum markers should be adjusted for maternal weight.

Our study had higher median values of PAPP-A, especially at older gestational ages and lower maternal weights, and lower median values of free β-hCG, at all gestational ages, and especially at higher maternal weights, when compared with the Caucasian-specific model [8]. However, our results also differed from a study in northern Thailand [9], which found significantly higher levels of both maternal serum markers compared to our study. The assays in the northern Thailand study [9] were performed using the DELFIA Xpress Immunoanalyzer System (Perkin Elmer, Waltham, MA, USA). The very high values of r for each prediction equation from northern Thailand [9] indicate that the equations were constructed by regressing the daily medians of the measured values against gestational age. Doing this, however, ignores the variability between subjects on each day that measurements are made. This can cause a bias in the estimations as each median carries the same weight in the regression, whereas in fact the days on which fewer measurements were made should carry lower weight than data from days with more measurements. Other possible explanations for the differences between that study [9] and ours, both in Thai women, are that the two populations comprise different ethnicities, or that different laboratory techniques led to different model equations. The optimal models for prediction may vary not only between countries but even among subpopulations with a single country.

Earlier studies have shown that adjustment for differing ethnicities increases the sensitivity of the test [6, 7]. However, this is achieved at the cost of an increased number of false positives which leads to more invasive procedures being conducted. Nevertheless, race-ethnicity specific adjustment is necessary for these biochemical markers in a first trimester screening program. In addition, the combination of serum biomarkers and the use of NT is recommended in risk estimation to give better specificity. As our center have defined normative data for NT thickness in each gestational week and the 95th percentile value for a given gestational age in the Thai population [13]. This study may add more sensitivity for first-trimester combined screening for trisomy 21 in our population.

Our study has several notable strengths. The data and findings of the study were derived from an unselected cohort of urban southern Thai women and biochemical analysis was performed immediately using a fresh sample of blood in a single laboratory, and using the standard analyzers. Furthermore, the quality of our laboratory was assessed on a monthly basis by the RIQAS Maternal Screening Program (MEDITOP CO., LTD., LONDON) and internal precision quality control. Another strength of the study was the completeness of the follow-up, which was done in every case. In particular, our model should be more accurate than both Caucasian and northern Thai models for identifying cases of trisomy 21 with borderline abnormal levels of serum PAPP-A and free β-hCG, because we used weight-adjusted equations based on each individual subject, which is the first such report from Thailand. The one notable limitation of the study was that it was conducted on a population of pregnant women attending at a single tertiary hospital in one part of southern Thailand. Another limitation was the relatively small sample size. If the screening program is started, the service system has to consider the counseling system, accuracy of maternal age (errors in the documentation of birth are common in Thailand due to cultural problems) and gestational age, quality of different laboratory techniques, and the option for diagnostic tests. Since our study was conducted in ‘urban’ women, a larger study including an appropriate amount of rural women is needed in the future.

## Conclusions

The study has successfully developed maternal-weight- and gestational-age-adjusted median normative models to convert the level of PAPP-A and free β-hCG to their MoM equivalents in southern Thai women. These models should have greater sensitivity than models based on other populations for first trimester trisomy 21 screening in our population.

## Supporting information

S1 Dataset Analysis file for this research. (XLSX)
